# Cellulose Nanofibrils/Alginates Double-Network Composites: Effects of Interfibrillar Interaction and G/M Ratio of Alginates on Mechanical Performance

**DOI:** 10.1021/acs.biomac.4c00093

**Published:** 2024-07-08

**Authors:** Li Zha, Finn Lillelund Aachmann, Håvard Sletta, Øystein Arlov, Qi Zhou

**Affiliations:** † Division of Glycoscience, Department of Chemistry, School of Engineering Sciences in Chemistry, Biotechnology and Health, KTH Royal Institute of Technology, AlbaNova University Centre, SE-106 91 Stockholm, Sweden; ‡ Norwegian Biopolymer Laboratory (NOBIPOL), Department of Biotechnology and Food Science, NTNU Norwegian University of Science and Technology, Sem Sælands vei 6/8, 7491 Trondheim, Norway; § Department of Biotechnology and Nanomedicine, SINTEF Industry, Richard Birkelands vei 3B, 7034 Trondheim, Norway

## Abstract

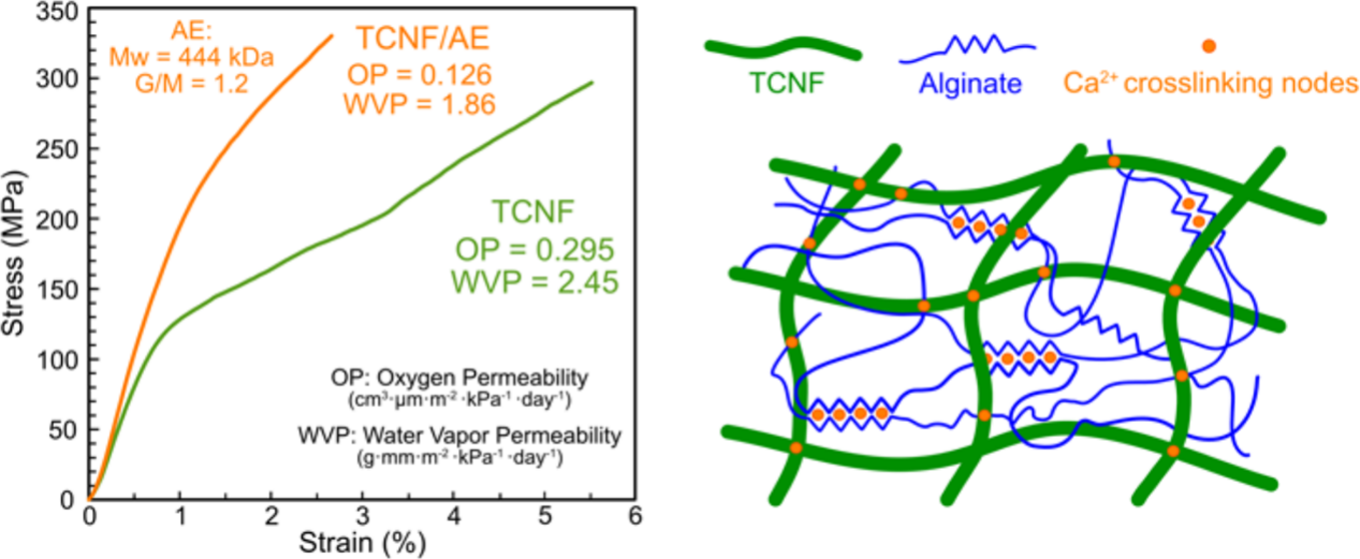

Interfibrillar phases and bonding in cellulose nanofibril (CNF)-based composites are crucial for materials performances. In this study, we investigated the influence of CNF surface characteristics, the guluronic acid/mannuronic acid ratio, and the molecular weight of alginates on the structure, mechanical, and barrier properties of CNF/alginate composite films. Three types of CNFs with varying surface charges and nanofibril dimensions were prepared from wood pulp fibers. The interfacial bonding through calcium ion cross-linking between alginate and carboxylated CNFs (TCNFs) led to significantly enhanced stiffness and strength due to the formation of an interpenetrating double network, compared to composites from alginates and CNFs with native negative or cationic surface charges. Various alginates extracted from *Alaria esculenta* (AE) and *Laminaria hyperborea* (LH) were also examined. The TCNF/AE composite, prepared from alginate with a high mannuronic acid proportion and high molecular weight, exhibited a Young’s modulus of 20.3 GPa and a tensile strength of 331 MPa under dry conditions and a Young’s modulus of 430 MPa and a tensile strength of 9.3 MPa at the wet state. Additionally, the TCNF/AE composite demonstrated protective properties as a barrier coating for fruit, significantly reducing browning of banana peels and weight loss of bananas stored under ambient conditions.

## 1 Introduction

Cellulose nanofibrils (CNFs) derived from various renewable resources have been extensively studied due to their intrinsic high aspect ratio, lightweight, and excellent mechanical properties, demonstrating significant potential for multifunctional biocomposites.^1,2^ To fully exploit this potential by engineering the interface in CNF-based materials, surface modifications are often performed on CNFs. These modifications include 2,2,6,6-tetramethyl-1-piperidinyloxy (TEMPO)-mediated oxidation, carboxymethylation, surface quaternization, lytic polysaccharide monooxygenase (LPMO) oxidation, and periodate oxidation.^3−9^ In both neat CNFs and CNF-based composites, the surface characteristics of CNFs dictate the interfibrillar phases and bonding, which are crucial for interfibrillar stress transfer and mechanical performance in both dry conditions and moist state.^10−12^

Alginates, commonly derived from brown algae, are highly charged block copolymers consisting of 1 → 4-linked α-l-guluronic acid (G) and β-d-mannuronic acid (M). The G/M ratio and block structure of alginates vary between algae species and even different parts of an alga. The adjacent G units can be cross-linked by divalent cations, typically Ca^2+^, usually described using an “eggbox” model.^13^ Previous studies have shown that the cross-linking conditions, G/M ratio, and molecular weights have a strong impact on the physicochemical properties of alginate materials. Films of alginate with a higher proportion of mannuronic acid than guluronic acid showed better mechanical properties.^14,15^ To enhance the stability and mechanical strength of hygroscopic alginate films, Ca^2+^ ion cross-linking and the addition of a plasticizer, such as glycerol, are often used. However, introducing plasticizers often compromises the tensile strength and network stiffness of Ca^2+^ cross-linked alginate.^16^

CNF/alginate composites have attracted significant research interest due to the unique properties of both alginate and CNFs. CNF/alginate composite aerogels and hydrogels have been utilized in biomedical applications, such as wound healing, drug release, and tissue engineering, where CNFs serve as a reinforcing network or scaffold to provide mechanical robustness and some ductility.^17−19^ Particularly, cross-linking alginate with divalent ions in cellulose/alginate composites has enabled the formation of supramolecular double-network materials consisting of a cellulose nanofiber network and a cross-linked alginate polymer network. Such composites exhibited remarkable wet integrity, improved absorption and encapsulation capacity, and enhanced barrier properties.^20−23^ However, the mechanical properties of cellulose/alginate composites are often compromised compared to those of neat cellulose materials. Cellulose has been used as the reinforcing component, but synergy of the double network is absent.

The interfacial interactions between cellulose and alginate are essential to unlocking the full potential of the double-network structure and properties of the composites. Therefore, understanding the impact of CNF surface characteristics on its interaction with alginates of various structures (G/M ratio and molecular weight) is crucial for designing CNF/alginate composites that benefit from a synergistic effect in the double-network structure, holding significant promise for broader applications. In this work, we investigated the effect of CNF surface characteristics on the water dispersion, structure, and mechanical properties of the CNF/alginate composites. Three different types of CNFs with various surface charges and nanofibril dimensions were prepared from commercial sulfite pulp using TEMPO-mediated oxidation, cellulase enzyme pretreatment, and cationic modification. Water dispersions of these CNFs with a commercial sodium alginate (SA) were characterized by UV–vis spectroscopy and dynamic light scattering (DLS) before the preparation of the CNF/SA composite films by solution casting, followed by calcium ion cross-linking. The carboxylated CNF (TCNF) obtained by TEMPO-mediated oxidation demonstrated a synergistic enhancement of the mechanical performance in the double-network structure with alginate. To further understand the influence of alginate structure on the properties of the composites, two types of alginates with different G/M ratios and molecular weights were extracted from cultivated *Alaria esculenta* (AE) and harvested *Laminaria hyperborea* (LH). The mechanical properties of the TCNF/AE and TCNF/LH composites were characterized in both the dry and wet states. The TCNF/AE composite, incorporating alginate of the highest molecular weight, was further applied as a barrier coating on fresh bananas to prevent browning and weight loss under ambient conditions, demonstrating potential in food packaging application.

## 2 Experimental Section

### 2.1 Materials

A commercially sourced, never-dried sulfite pulp from softwood was supplied by Nordic Paper, Sweden, and used to prepare CNFs with different types of surface charges. Sodium hypochlorite (NaClO), sodium bromide (NaBr), sodium hydroxide (NaOH), 2,2,6,6-tetramethyl-1-piperidinyloxy (TEMPO), glycidyltrimethylammonium chloride (GTMAC), silver nitrate (AgNO_3_), calcium chloride (CaCl_2_), sodium alginate (SA) from brown algae (Sigma, Lot #SLBV4496, medium viscosity, MW 250–350 kDa, 60–70% M, 30–40% G, M/G ratio = 1.56), and all other chemicals were purchased from Merck KGaA, Germany, and used as received.

### 2.2 Preparation of CNFs

TEMPO-oxidized CNFs (TCNFs) were prepared from the wood pulp fibers by using the NaClO/NaBr/TEMPO oxidation system at pH 10.^24^ The TEMPO-oxidized pulp suspension was mechanically disintegrated to obtain a 1 wt % TCNF water dispersion (pH = 6.7–6.8) by using a kitchen blender (Vita-Prep 3 model, Vita-Mix Corp.). The carboxylate content of TCNFs was determined by the conductometric titration method reported previously.^25^ The CNFs from enzyme-pretreated pulp (EnzyCNF) were prepared by using a commercial monocomponent endoglucanase (Novozym 476, 5000 ECU/g, Novozymes A/S, Denmark) with an enzyme loading of 5 μL/g wood pulp fibers. Subsequently, a 2 wt % water suspension of the enzyme-pretreated pulp fibers (pH = 6.8–6.9) was mechanically disintegrated by using a mircrofluidizer (M-110EH, Microfluidics) to produce EnzyCNF.^26,27^ The content of the negative charge in the obtained EnzyCNF was also measured by conductometric titration. CNFs carrying cationic quaternary ammonium-based molecules on their surfaces (QCNF) were prepared by chemical modification of wood pulp fibers via nucleophilic addition reaction using GTMAC as described in our previous work.^5^ The quaternized wood pulp fibers were subsequently dispersed in water at a solid content of 0.5 wt % and disintegrated by the kitchen blender to produce QCNFs (pH = 6.9–7.0). The content of the quaternary ammonium cation was estimated from the number of trimethylammonium chloride groups obtained by conductometric titration of chloride ions, assuming the presence of one chloride counterion per trimethylammonium group.^5^ Typically, 100 mg of QCNF sample was dispersed in 100 mL of ultrapure water and titrated with an 8 mM AgNO_3_ aqueous solution by adding 0.2 mL in 60 s intervals. The amount of trimethylammonium groups was calculated based on the volume of AgNO_3_ used in the titration.

The intrinsic viscosity numbers of the CNF samples were measured according to the ISO 5351:2000 standard. Typically, 30 mg of CNFs was dissolved in 50 mL of 0.5 M copper ethylenediamine (CED) for 30 min and measured with a capillary viscometer. The degree of polymerization (DP) was calculated from intrinsic viscosity data, η, using the equation η = 0.42DP for the EnzyCNF and QCNF samples, the equation η = 2.84DP^0.67^ for the TCNF sample, and the equation η = 2.28DP^0.76^ for the original pulp fibers.^27−29^

### 2.3 Extraction and Characterization of Alginates (AE1, AE2, AE3 and LH1, LH2, LH3)

In addition to the commercial Sigma SA, AE alginates were extracted from frozen cultivated *A. esculenta* provided by Seaweed Solutions AS, Norway. The seaweed biomass was thawed, milled, and split into three batches before the addition of 0.2 M HCl to each of the batches (2 L/kg biomass). The batches were incubated under shaking (200 rpm, orbital movement 2.5 cm amplitude) at 50 °C for 6, 12, or 20 h to produce alginates (AE1, AE2, AE3) with different molecular weights. The biomass was centrifuged (3220*g*, 15 min) and washed once with deionized water before adding 0.2 M NaHCO_3_ (4 L/kg biomass) to all batches, adjusting the pH to 7. The batches were incubated at 20 °C for 20 h under shaking (200 rpm, orbital movement of 2.5 cm amplitude), followed by centrifugation as described above. Alginate was precipitated from the supernatant by adjusting the pH to 2 using 3 M HCl, followed by washing with 0.05 M HCl, 50% ethanol, 70% ethanol, and 100% ethanol (once per treatment). The alginate was finally lyophilized before analysis. The LH alginates (LH1, LH2, and LH3) originated from the stipes of wild-harvested *L. hyperborea* and were supplied by Dupont Nutrition Norway AS.

### 2.4 Preparation of CNF/Alginate Composites

The CNF/alginate composite films were prepared by solution casting, followed by ionic cross-linking. Typically, a 50 mL water suspension containing 100 mg of CNFs and 100 mg of alginate was mixed by an Ultra-Turrax disperser (IKA T25) for 2 min at 20,000 rpm, followed by degassing and solution casting in a Petri dish (Sarstedt, (Ø × *H*): 92 × 16 mm, material: PS) and drying under ambient conditions at 22 °C. The dry films were soaked in 0.1 M CaCl_2_ solution for 1 h. The wet films were thoroughly rinsed with deionized water before drying in a Rapid Köthen sheetformer under vacuum pressure at 93 °C for 5 min. Neat CNFs and neat alginate films were prepared in the same manner. The thickness of the films was 20–23 μm. The density of the films was calculated based on the measured dry sample weight and dimensions.

### 2.5 Characterizations

The molecular weights of the AE and LH alginates were analyzed at room temperature on an HPLC system fitted with an OHpak LB 806 M size exclusion column using 0.15 M NaNO_3_ and 0.01 M EDTA, pH 6.0, as an eluent at a flow rate of 0.5 mL/min. The column outlet was connected to a Dawn Helios II multiangle laser light scattering photometer (Wyatt) (λ_0_ = 663.8 nm) and a Shodex RI-501 refractive index detector. Data were collected and processed using ASTRA software v. 7.3 (d*n*/d*c* = 0.150 mL/g and *A*_2_ = 5.0 × 10^–3^ mL·mol·g^–2^). All samples were run as triplicates.

G/M ratio of the alginates was determined by ^1^H NMR. The alginate samples underwent a mild acid hydrolysis to achieve an approximate degree of polymerization (DP) of 70 before collecting NMR data, as previously described.^30−32^ Approximately 10 mg of the hydrolyzed samples was dissolved in 600 μL of D_2_O (d-99.9%, Sigma-Aldrich) and left to dissolve overnight. Triethylenetetramine-hexaacetic acid (TTHA) in D_2_O (0.3 M, 20 μL) was introduced as a chelator to remove residual cations. After centrifugation, the supernatants were transferred to NMR tubes. To provide an internal chemical shift reference, 3-(trimethylsilyl)-propionic-acid sodium salt (TSP) (Aldrich, Milwaukee, WI) in D_2_O (1%, 5 μL) was added. ^1^H NMR spectra were recorded at 83 °C on a BRUKER NEO 600 MHz instrument equipped with a 5 mm iProbe (Bruker BioSpin). The spectra were recorded using TopSpin 4.0.8 software (Bruker BioSpin) and processed and analyzed with TopSpin 4.0.7 software (Bruker BioSpin).

Monosaccharide composition of the wood pulp fibers and CNFs was characterized by carbohydrate analysis. Typically, 200 mg of the sample was hydrolyzed with sulfuric acid, and the monosaccharide content of the acid hydrolysate was quantified by a Dionex ICS-6000 ion chromatography system (Thermo Fisher Scientific Inc.) using a Dionex CarboPac PA1 column. The XRD diffractograms of TCNF, EnzyCNF, and QCNF were recorded with a Philips X’Pert Pro diffractometer (model PW 3040/60) in the reflection mode. The Cu Kα radiation (λ = 1.5418 Å) was generated at 45 kV, 40 mA, monochromatized with a 20 μm Ni filter. High-resolution AFM images of the TCNFs, EnzyCNFs, and QCNFs were recorded using ScanAsyst mode on a MultiMode 8 AFM system (Bruker, Santa Barbara, CA). The samples were prepared by drying a 10 μL droplet of diluted CNF suspension on a silica wafer that was pretreated on a PELCO easiGlow glow discharge cleaning system. The resonance frequency of the cantilever is 70 kHz, and the nominal tip radius is 2 nm, with a spring constant of 0.4 N/m. The optical transmittance of the water suspensions (0.1 wt %) of CNFs, SA, and their mixtures (50/50 by weight) were characterized by UV–vis spectroscopy (Varian Cary 50 Bio) in a wavelength range of 400–1000 nm. The CNF suspensions were also characterized by dynamic light scattering (DLS, Zetasizer nano ZS, Malvern, Worcestershire, U.K.) to measure hydrodynamic size distributions and ζ-potential.

The freeze-fractured composite films were coated with gold–palladium using a Cressington 208HR sputter coater before imaging with field-emission scanning electron microscopy (FE-SEM, Hitachi S4800) operated at 1 kV. Tensile testing of the composite films was performed by using an Instron 5944 Universal Testing Machine equipped with a 500 N load cell. The width of the specimen was 3 mm, and the gauge length was 20 mm. The samples were conditioned at 50% relative humidity (RH) for 2 days prior to the measurement. The strain rate was 10% per minute, and an advanced video extensometer was used to measure the tensile strain. For each sample, at least 5 specimens were measured. For the measurement of mechanical properties at the wet state, samples were cut and soaked in ultrapure water for at least 24 h prior to the tensile testing. The thickness swelling in percentage was calculated by subtracting the dry thickness from the wet thickness at 24 hours and then dividing it by the dry thickness to evaluate the network integrity at the wet state.

Water vapor permeation of the TCNF and TCNF/AE1 composite films was measured gravimetrically at 23 °C, 50% RH. Typically, a 100 mL glass vial containing 10 g of anhydrous calcium chloride was sealed with a film sample secured by a screw cap with a hole. The film sample was surrounded by using an aluminum mask with an exchange surface area of 5 cm^2^ to tightly close the glass vial with the screw cap. The test vials were placed under room conditions at 23 °C, 50% RH, and weighed at time intervals of 24 h until the weight increase reached a constant value. Three films were measured for each sample. The water vapor transmission rate (WVTR) was calculated based on the measured amount of water vapor mass, the exposed film area, and the testing time.

The oxygen permeability was measured at 22 °C and 30% RH using a MOCON OX-TRAN 2/22 10X model (Minneapolis, MN). The sample film area was 5 cm^2^ and the flow rate of oxygen through the film was measured. From the steady-state flow rate, the oxygen transmission rate (OTR) was calculated. Two measurements were made for each sample.

The TCNF/AE1 composite was demonstrated as a barrier coating onto fresh bananas using an RS PRO Air Brush spray gun (AB931, 0.3 mm) to reduce browning and weight loss under ambient conditions. Nontreated bananas were used as the control. Photos and weight loss of the bananas were recorded at different time intervals to evaluate the impact of the surface coating.

## 3 Results and Discussion

### 3.1 Water Dispersions of the CNF/SA Mixtures

**Figure 1:**
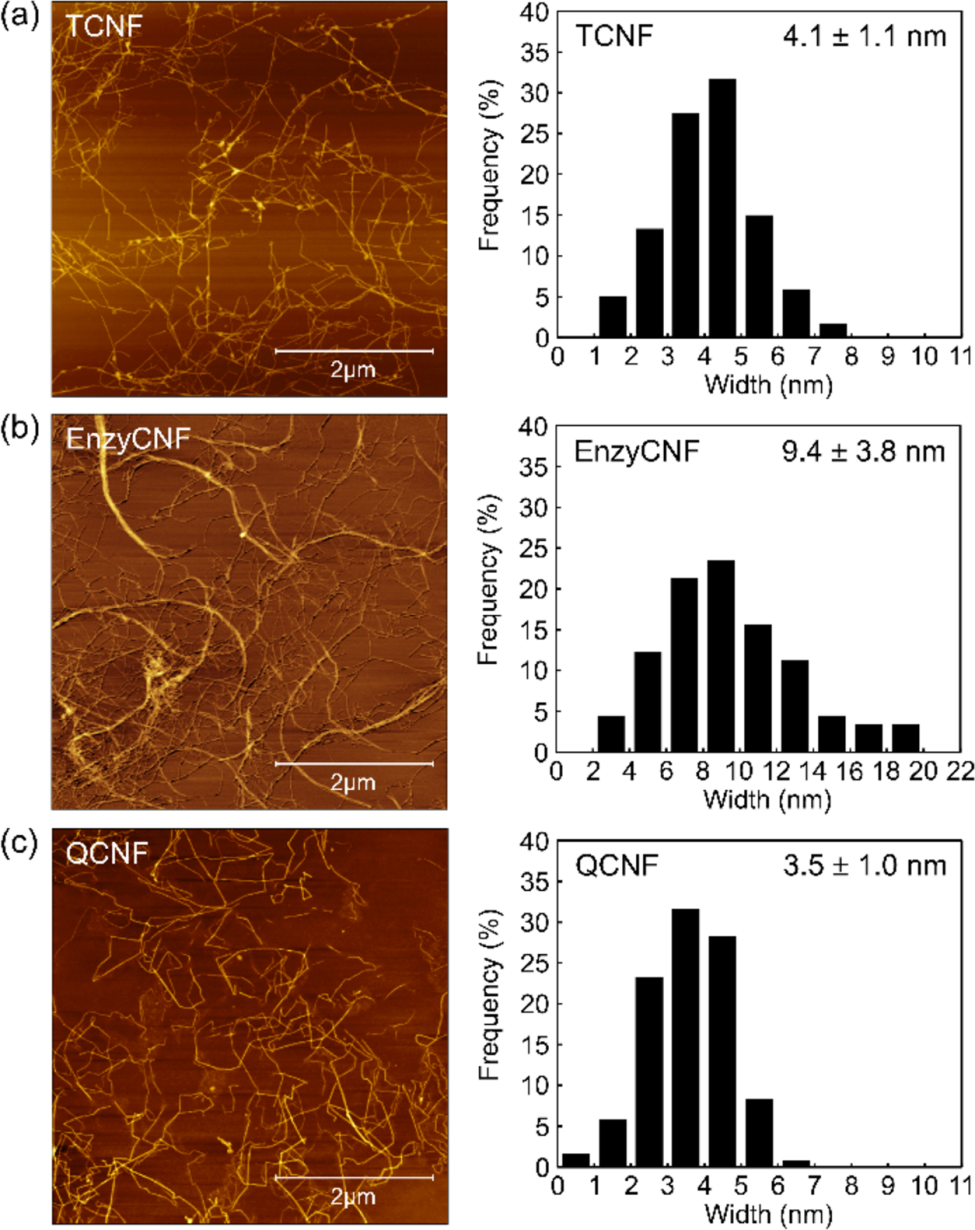
AFM height images of (a) TCNF, (b) EnzyCNF, and (c) QCNF and the corresponding histograms of their width distributions.

TCNF with a carboxylate content of 1.50 mmol/g, EnzyCNF with a negative charge of 0.13 mmol/g, and QCNF with a trimethylammonium group content of 1.80 mmol/g were successfully prepared from wood pulp fibers. The hemicellulose content of the native wood fiber was 18.1%, as determined by sugar analysis (Table S1). After enzymatic or chemical modification, the hemicellulose content of EnzyCNF, TCNF, and QCNF decreased to 14.0, 9.4, and 3.2%, respectively. Figure 1 shows AFM height images of the diluted suspensions of TCNF, EnzyCNF, and QCNF dried on a silica wafer, along with the corresponding histograms of width distributions, which were measured by using the heights of the nanofibrils by image analysis. Both TCNF and QCNF showed a morphology of well-individualized discrete cellulose nanofibrils due to their high surface charges. The average width of TCNF was 4.1 ± 1.1 nm, while the average width of QCNF was 3.5 ± 1.0 nm. The EnzyCNF showed broader width distribution and the average width was 9.4 ± 3.8 nm. The QCNF, TCNF, and EnzyCNF samples showed a typical XRD diffraction pattern of cellulose I, same as the native wood pulp fibers (Figure S1). The two peaks centered at 14.8 and 16.8° in the X-ray diffraction patterns were separated by curve fitting. The crystal sizes of the corresponding planes with *d*-spacing of 0.60–0.61 and 0.53–0.54 nm were calculated from full widths at half heights of the diffraction peaks by Scherrer’s equation. The average crystal size of native wood pulp fiber was 4.1 nm, while the average crystal sizes of TCNF, QCNF, and EnzyCNF were 2.7, 2.4, and 2.9 nm, respectively, indicating that chemical and enzymatic modifications reduce the crystallite size of cellulose. In addition, the DP values for TCNF, EnzyCNF, and QCNF were 450, 930, and 360, respectively, significantly lower than that for the wood fiber (DP of 1870). The crystallinity index (CI) of cellulose was calculated from the content of amorphous cellulose, which was estimated using the ratio between the intensity of the minimum between the 200 and 110 peaks and the intensity of the 200 peak.^7^ The CI values of cellulose for TCNF, EnzyCNF, and QCNF were 70.7, 73.1, and 63.2%, respectively, while the CI of cellulose for the native wood pulp fibers was 67.5%.

**Figure 2:**
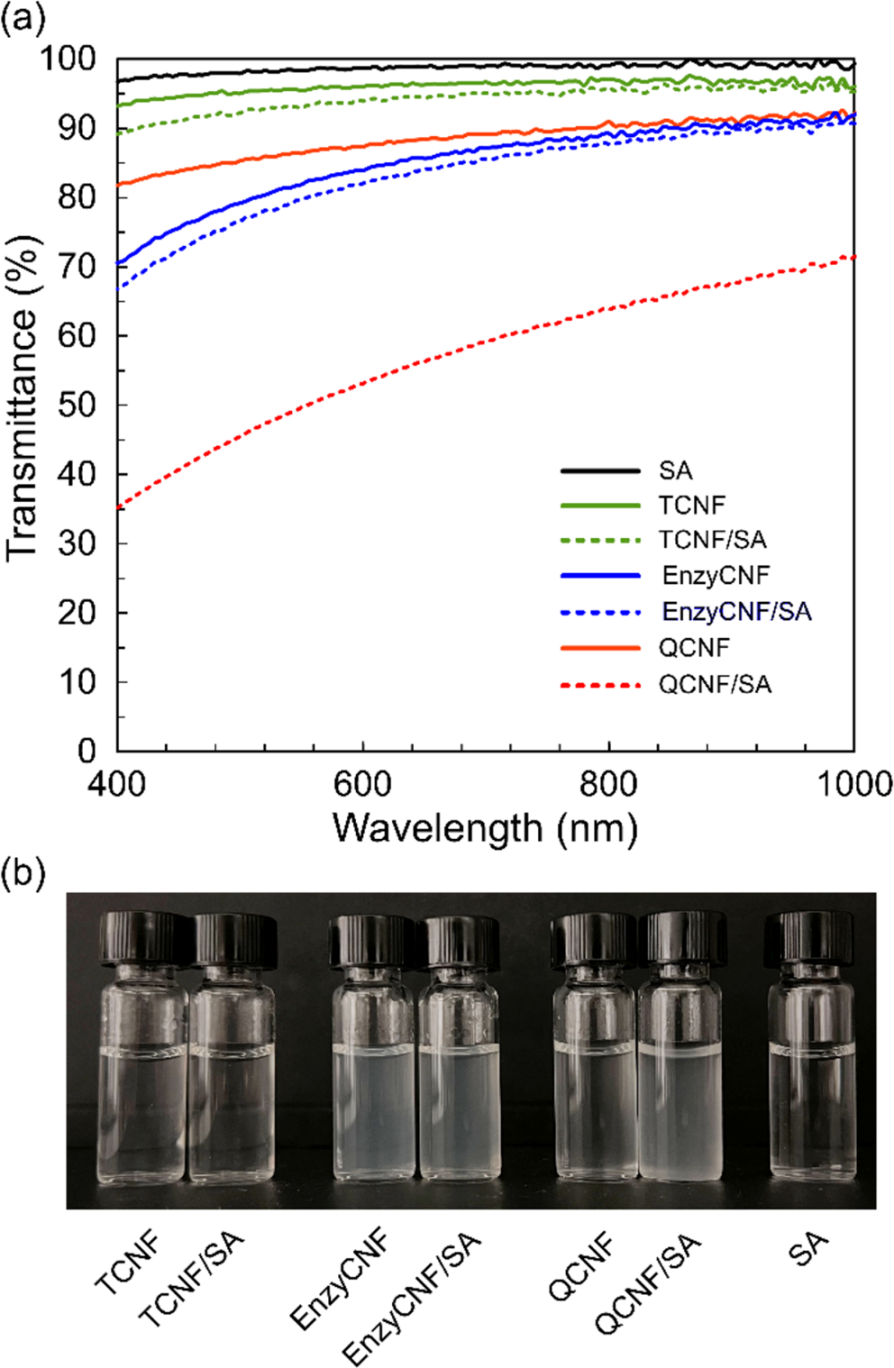
(a) Light transmittance of 0.1 wt % water dispersions of SA, different CNFs, and the corresponding CNF/SA mixtures and (b) the photographs of their water dispersions.

The interactions between SA and different CNFs in the water suspensions were characterized by UV–vis spectroscopy. The light transmittance of water dispersions of TCNF, EnzyCNF, QCNF, and their mixtures with SA are shown in Figure 2a. Usually, a higher optical transmittance indicates a thinner nanofiber width in the CNF suspension, as larger fiber clusters would cause light scattering and elevated turbidity.^33^ The SA solution showed a high light transmittance of 99% at 600 nm due to the complete dissolution of SA in water, while the light transmittance of the neat TCNF suspension was 95% at 600 nm due to well-individualized thin cellulose nanofibers. The light transmittance of the TCNF/SA mixture was maintained at 94% at 600 nm, indicating that the TCNF nanofibers were kept discrete in the presence of SA due to the electrostatic repulsion between TCNF and SA. Indeed, the ζ-potentials of SA water solution and TCNF and TCNF/SA water suspensions were −72.5 ± 0.7, −60.8 ± 1.1, and −66.4 ± 1.2 mV, respectively. A similar phenomenon was observed for EnzyCNF, where the light transmittance of EnzyCNF water dispersion decreased slightly from 84 to 82% at 600 nm after mixing with SA. The EnzyCNF was slightly negatively charged (ζ-potential of −35.2 ± 1.6 mV) due to the presence of residual hemicelluloses, while the EnzyCNF/SA suspension showed a ζ-potential of −55.4 ± 2.1 mV.

In contrast, strong ionic interactions between positively charged QCNF and negatively charged SA resulted in increased turbidity in the water suspension (Figure 2b). The light transmittance of the QCNF/SA mixture was 53% at 600 nm, significantly lower than that (87%) for the water dispersion of neat QCNF. Such a phenomenon was reported previously that when mixing QCNF with negatively charged nanoclay (sodium montmorillonite), large aggregates were formed and the optical transmittance of the composite suspension decreased accordingly.^11^ The ζ-potential of the QCNF water suspension was 64.0 ± 2.6 mV, while a negative value of −32.3 ± 1.0 mV was obtained for the QCNF/SA mixture. Therefore, besides the SA that ionically interacted and bound the adjacent CNFs, there was excessive SA, which contributed to a stable colloidal QCNF/SA suspension. The theoretical charge screening point was not desirable for composite preparation as it would bring the ζ-potential of the suspension close to zero, leading to the formation of large aggregates due to the insufficient surface charges of the colloidal particles in the dispersion.^34^

**Figure 3:**
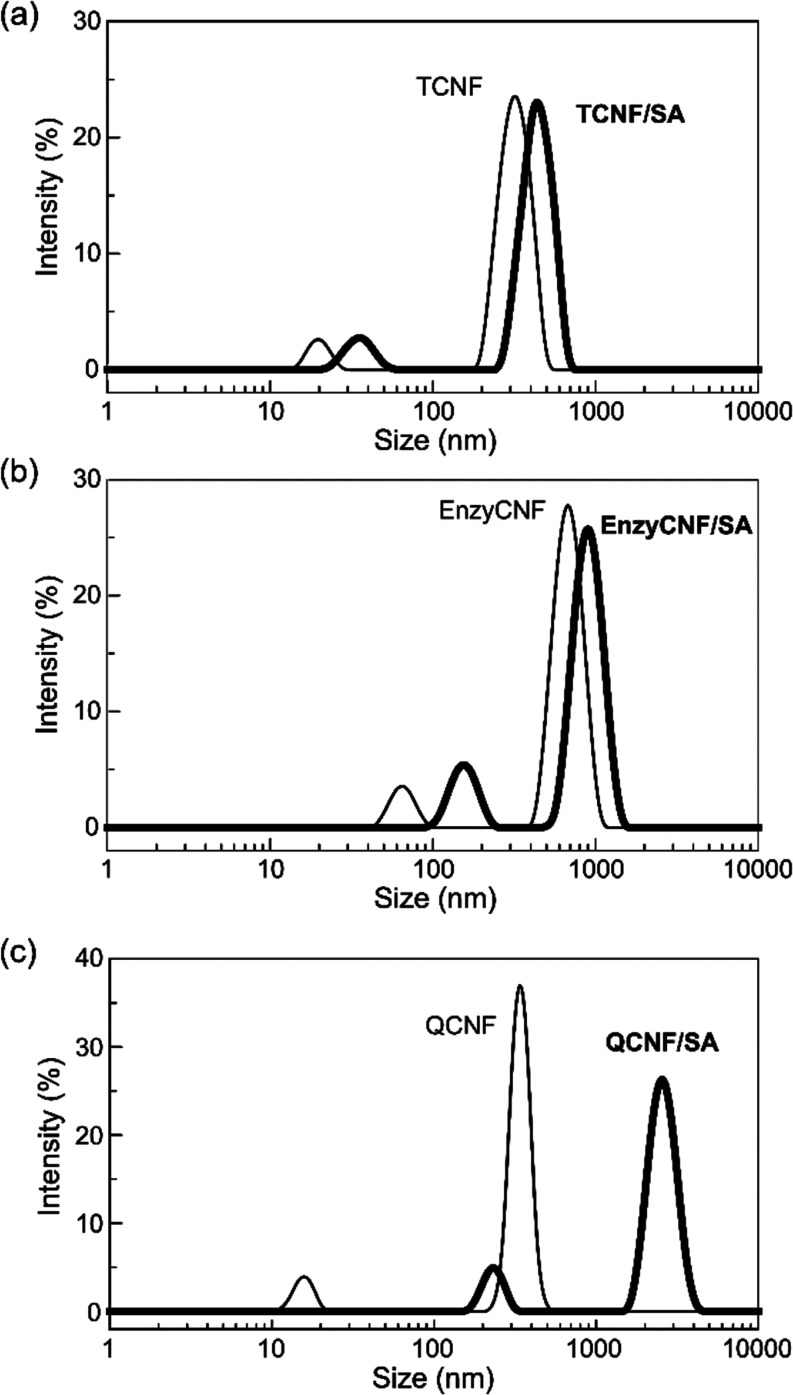
Estimated hydrodynamic size distributions of the cellulose nanofibers in the 0.1 wt % water suspensions of (a) TCNF and TCNF/SA, (b) EnzyCNF and EnzyCNF/SA, and (c) QCNF and QCNF/SA.

The formation of large fibril aggregates in the suspension of the QCNF/SA mixture was further confirmed by changes in particle size (hydrodynamic diameter) as monitored by DLS. Figure 3 shows the estimated hydrodynamic size distributions of the three different types of CNFs before and after mixing with SA. All CNF samples presented bimodal distribution of hydrodynamic size, typically observed for cellulose nanofibers in water suspensions.^35,36^ The experimentally determined hydrodynamic diameter does not reflect the dimensions of CNFs as measured from AFM and XRD. However, it can reveal a decrease in particle mobility as a consequence of, for example, irreversible aggregation.^37^ The *Z*-average hydrodynamic diameters (*D*_h_) of TCNF, EnzyCNF, and QCNF nanofibers were 324.0 ± 26.5, 637.4 ± 40.9, and 312.3 ± 19.2 nm, respectively. After mixing with SA, the *D*_h_ of TCNF and EnzyCNF increased slightly to 473.2 ± 54.4 and 803.9 ± 79.1 nm, respectively, possibly due to the chain entanglement of the SA molecules on the surface of CNFs. The *D*_h_ of QCNF increased 6.6 times to 2369.1 ± 202.7 nm when mixed with SA, indicating the formation of irreversible large fibril aggregates due to the dominant ionic interaction between QCNF and the SA.

### 3.2 Structure and Mechanical Properties of the CNF/SA Composites

The FTIR spectrum of QCNF showed a band at 1480 cm^–1^, corresponding to the trimethyl groups of quaternized ammonium (Figure S2). The FTIR spectrum of TCNF showed a strong band at 1599 cm^–1^, corresponding to the carboxylate group in the salt form. A band at 1586 cm^–1^ was observed in the SA sample due to the presence of carboxylate groups cross-linked by Ca^2+^. For the QCNF/SA, EnzyCNF/SA, and TCNF/SA composites, the peak positions of carboxylate groups were 1598, 1595, and 1591 cm^–1^, respectively. Strong interactions with multivalent ions in the coordination bonds change the vibration energies for carboxylate groups and result in the asymmetric vibration shifting toward lower wavenumbers.^38^ These results indicated that the coordination level of the carboxylate group was higher in the TCNF/SA composite, which contributed to the formation of a double network in the composites.

**Figure 4:**
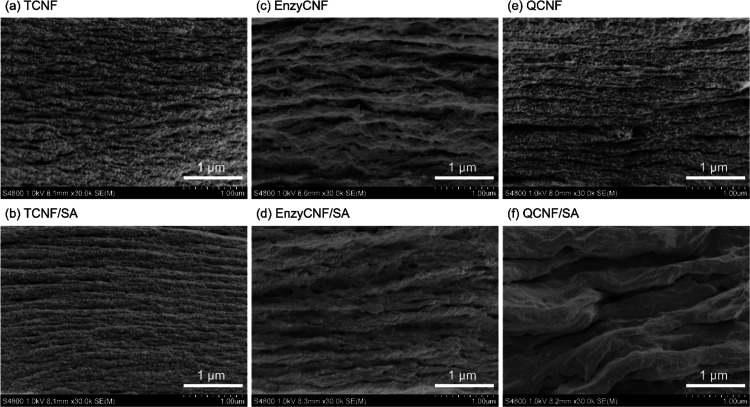
FE-SEM images for the freeze-fractured cross sections of the neat CNFs and the CNF/SA composite films: (a) TCNF, (b) TCNF/SA, (c) EnzyCNF, (d) EnzyCNF/SA, (e) QCNF, and (f) QCNF/SA.

The morphological structure of the TCNF/SA, EnzyCNF/SA, and QCNF/SA composites was characterized by using SEM on the freeze-fractured cross sections (Figure 4) and the surfaces (Figure S3) of the films. The neat TCNF film presented a homogeneous porous network composed of finely individualized nanofibril layers (Figure 4a). The Ca^2+^ ion can effectively cross-link negatively charged TCNF and increase the density and barrier property of the TCNF network.^39^ Interestingly, after incorporation with SA, the TCNF nanofibril network was less porous, rather denser and smoother layers were obtained (Figure 4b). This can be attributed to the interfacial cross-linking between TCNF and SA facilitated by the Ca^2+^ treatment. Such interfacial Ca^2+^ ion cross-linking resulted in the formation of an interpenetrating double network with strong interfacial bonding between the cross-linked SA polymer network and the entangled TCNF nanofibril network. The cross section of the EnzyCNF film (Figure 4c) showed thicker fiber layers that were formed during the drying process due to aggregations and large voids were obvious between the deposited layers.^40^ After the addition of SA, the EnzyCNF/SA composite showed a similar thick fiber layer structure with spotted features that were resulted from Ca^2+^ cross-linking (Figure 4d). Previous study has reported a similar phenomenon on the surface morphology, where more spotted and rougher morphology was observed when neat alginate films were cross-linked with a higher Ca^2+^ concentration.^15^ The neat QCNF film showed fibril layers with sizes comparable to TCNF, but minor gaps were present on the cross sections, compromising the network density (Figure 4e). In the QCNF/SA composite, a distinctly different morphology emerged, characterized by even thicker layers consisting of large fibril bundles, in line with the large aggregates as observed in the QCNF/SA water suspension.

**Figure 5:**
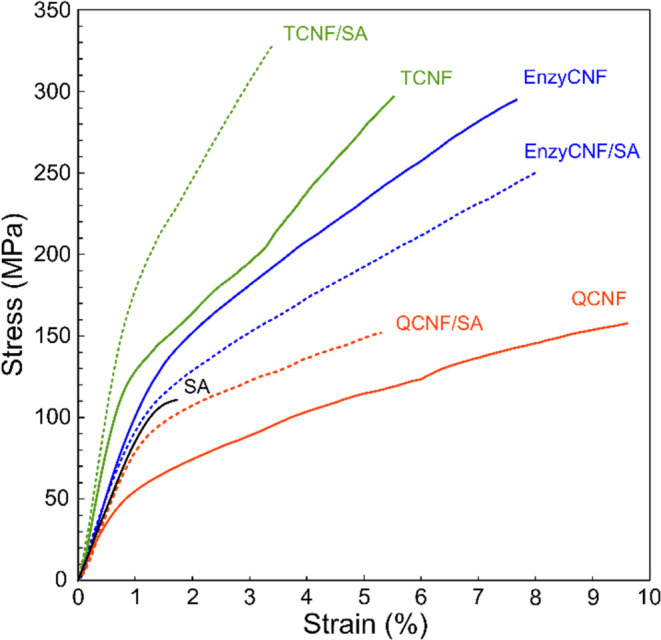
Typical tensile stress–strain curves of the neat CNFs, neat SA, and CNF/SA composite films at 50% RH.

Figure 5 shows typical tensile stress–strain curves of the neat CNFs, neat SA, and CNF/SA composite films at 50% RH and their mechanical properties are summarized in Table S2. The neat SA film showed rather high stiffness with a Young’s modulus of 8.5 GPa yet brittle with a strain to failure of 1.8%, comparable with previous studies.^15,20^ The neat QCNF film presented a Young’s modulus of 7.3 GPa and a tensile strength of 158 MPa, lower than those for the EnzyCNF and TCNF films, mainly due to its lower density (1.32 g/cm^3^) and lower DP of cellulose (360). The yield strength of the QCNF/SA composite film increased by 80% compared to the neat QCNF due to the formation of larger fiber aggregates through ionic interactions. However, this structural change, along with the presence of local pores, did not lead to a significant increase in the overall density of the composites (from 1.32 to 1.34 g/cm^3^). Instead, it compromised the ability for fibril slippage, leading to earlier breakage during tensile deformation. Additionally, the slope in the plastic region remained almost identical to that of neat QCNF, indicating that the resistance to fibril slippage did not improve. Such mechanical performance indicated that while the SA successfully cross-linked the QCNF to form larger fibril aggregates, it did not contribute to the enhancement of tensile strength or toughness of the composite.

The neat EnzyCNF film with a density of 1.37 g/cm^3^ and cellulose DP of 930 showed a Young’s modulus of 10.4 GPa and a tensile strength of 295 MPa. This sample had a lower modulus and higher tensile strength than the cellulose nanopaper structure of EnzyCNF with a density of 1.22 g/cm^3^ and a cellulose DP of 800, which showed a Young’s modulus of 14.7 GPa and a tensile strength of 205 MPa.^27^ This is due to the higher hemicellulose content (18.1%) of EnzyCNF in this work as compared to that (13.8%) in the literature. However, upon incorporation of SA, the stiffness, yield strength, and ultimate strength of the EnzyCNF/SA composite all significantly decreased. The addition of SA did not substantially increase the density of the composite. Without interfacial cross-linking, the Ca^2+^ only cross-linked the SA phase in the composite, potentially creating local weak spots. This, in turn, weakened the interfibrillar bond between EnzyCNFs and resulted in slightly plasticized mechanical behavior, a phenomenon often observed in CNF/polysaccharide composites, such as CNF/xyloglucan,^41,42^ CNF/starch,^35^ and CNF/CMC.^40^

The neat TCNF film with a density of 1.41 g/cm^3^ and a cellulose DP of 450 exhibited higher mechanical properties than the EnzyCNF film, with a Young’s modulus of 14.3 GPa, a yield strength of 118 MPa, and a tensile strength of 300 MPa. This is similar to mechanical properties of the TOCN film with a density of 1.43 g/cm^3^ and a cellulose DP of 400 reported previously, which showed a modulus of 9.8 ± 0.8 GPa and a tensile strength of 266 MPa without calcium ion cross-linking.^43^ When the TCNF and SA were integrated with cross-linking at their interfaces, the composite showed an increased density of 1.48 g/cm^3^, a Young’s modulus of 20.0 GPa, a yield strength of 166 MPa, and a tensile strength of 327 MPa. In contrast, based on the rule of mixture, using the volume fraction and modulus of TCNF (*V*_f_ of 49.3%, *E* of 14.3 GPa) and SA (*V*_f_ of 46.3%, *E* of 8.5 GPa), the modulus of the TCNF/SA composite is calculated to be only 11.0 GPa. Such synergistic enhancement in mechanical performance was not observed in the EnzyCNF/SA composite, where although the double networks were formed, the interfacial cross-linking between the CNFs and the SA networks was absent. Moreover, the clearly improved resistance to fibril slippage, indicated by the higher slope in the plastic region of the TCNF/SA composite, was attributed to the interlocking effect resulting from interfacial cross-linking within the double networks. Therefore, TCNF was chosen for further study and application demonstration due to its remarkable ability to form desirable interpenetrating double-network composites with alginates. The Young’s modulus and tensile strength of TCNF/SA composites were higher than those of CNC/alginate composites (10.93 GPa, 41.3 MPa)^44^ and CNF/alginate composites (7 GPa, 140 MPa)^45^ in previous studies. Additionally, TCNF/SA composites also exhibited superior mechanical properties compared to other ionically cross-linked double-network composites in the literature, including carboxymethylated CNF/alginate (10.5 GPa, 300 MPa)^20^ and dicarboxylic acid CNF/alginate composites (17 GPa, 125.31 MPa).^23^

### 3.3 Effect of G/M Ratio and Molecular Weight of Alginates

To investigate the effect of the G/M ratio and molecular weight of alginates on the properties of TCNF/alginate composites, an array of alginate samples was obtained and prepared from cultivated (AE) and wild (LH) seaweeds. NMR characterization showed a higher guluronic acid content in LH alginates (*F*_G_ = 0.66–0.69) compared with AE alginates (*F*_G_ = 0.54–0.57), as well as higher fractions and the average length of G-blocks, which has already been established in previous studies.^46,47^ Increasing the time of the acid pretreatment of the seaweed biomass at a relatively high temperature (50 °C) was found to reduce the molecular weight of the extracted alginates (Table 1). As the LH alginates were provided by an external supplier, the production conditions were not known, but the analysis showed varying MW for the three selected samples.

**Table 1:** Average Fraction of Guluronic Acid (*F*_G_), Mannuronic Acid (*F*_M_), Single Guluronic Acids (*F*_MGM_), Triad of Guluronic Acid (*F*_GGG_), the Average Length of G-blocks (N_G > 1_), and Weight Average Molecular Weight (MW) of the Alginates

sample	*F*_G_	*F*_M_	*F*_MGM_	*F*_GGG_	*N*_G > 1_	MW (kDa)
SA	0.37	0.63	0.15	0.14	4	304
AE1	0.54	0.46	0.09	0.37	11	444
AE2	0.54	0.46	0.10	0.37	12	230
AE3	0.57	0.43	0.08	0.40	11	145
LH1	0.69	0.31	0.07	0.53	14	267
LH2	0.66	0.34	0.09	0.48	12	202
LH3	0.68	0.32	0.08	0.52	14	107

The typical tensile stress–strain curves for the films of the SA, AE, and LH alginates are compared in Figure S4. The AE and LH alginate samples were also brittle, with a strain to failure of less than 2.1%. Their stiffness, due to Ca^2+^ cross-linking, was relatively high, with a Young’s modulus ranging from 7.7 to 12.4 GPa. The neat AE and LH films showed slightly higher density and Young’s modulus with higher molecular weights. Low-molecular-weight AE3 (145 kDa) and LH3 (107 kDa) primarily exhibited elastic deformation, while higher-molecular-weight AE and LH samples also presented extended plastic deformation. When comparing SA, AE2, and LH1 of similar molecular weight close to 250 kDa, the LH1 film showed higher Young’s modulus (9.4 GPa) while the SA film showed higher tensile strength (115 MPa). The higher Young’s modulus of LH is related to its higher guluronic acid content, which leads to higher Ca^2+^ cross-linking density, while the higher tensile strength of SA might be due to its higher mannuronic acid content, as previous study has shown that M-rich blocks can serve as mediators and promote the self-assembly of alginate chains.^48^

**Figure 6:**
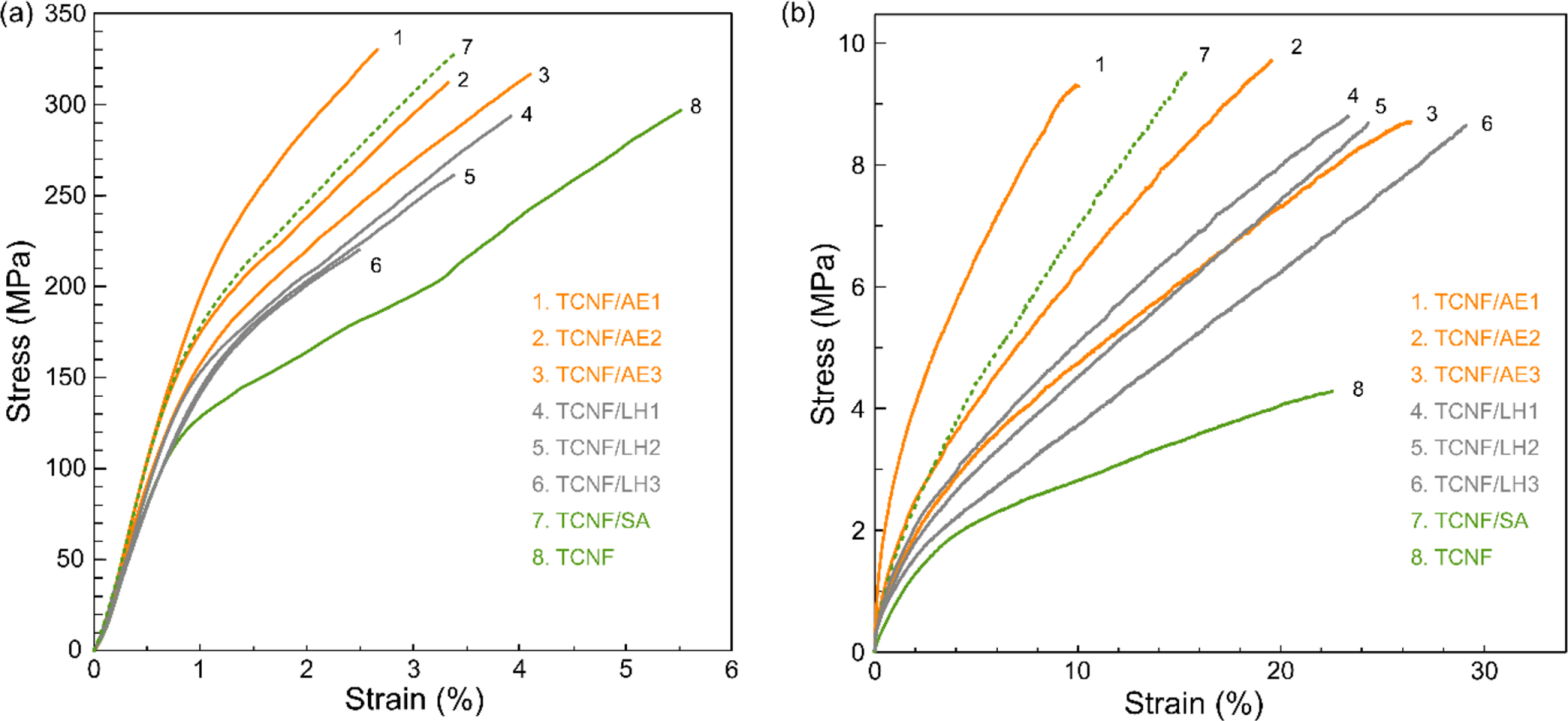
Typical tensile stress–strain curves of the TCNF/AE and TCNF/LH composite films compared to the neat TCNF and the TCNF/SA composite at the (a) dry state (RH50%) and (b) wet state.

Figure 6a shows typical tensile stress–strain curves of the neat TCNF and different TCNF/alginate composites at the dry state under 50% RH, and their mechanical properties are summarized in Table S3. When comparing the TCNF/AE and TCNF/LH composite films with the neat TCNF film, all composites showed synergistic enhancement in mechanical properties despite the inherent brittleness of neat AE and LH alginates, indicating stronger composite network through interfacial cross-linking, same as the TCNF/SA composite. Notably, the overall composite density, yield strength, and slope of plastic deformation were similar for the TCNF/LH1, TCNF/LH2, and TCNF/LH3 samples. Increasing molecular weight of LH mainly led to higher tensile strength and strain to failure, suggesting that the molecular weight of LH did not significantly alter the interfacial interaction between TCNF and LH. For the TCNF/AE composites, an increase in molecular weight of AE led to a significant increase in Young’s modulus, yield strength, and the slope of plastic deformation, while the strain to failure decreased accordingly. This suggested stronger interfacial interactions between TCNF and AEs as the molecular weight increased. The molecular weight of alginate is a dominant factor as the TCNF/AE1 composite demonstrated the highest stiffness and strength.

When comparing the TCNF/SA, TCNF/AE2, and TCNF/LH1 composites with similar alginate molecular weights close to 250 kDa, the TCNF/SA and TCNF/AE2 composites exhibited higher increases in Young’s modulus and tensile strength. One plausible explanation was that the SA and AE alginates were rich in mannuronate units, making their extended conformation proportion dominant. During calcium ion cross-linking, TCNF/alginate already formed a network in the composite nanopaper. To achieve optimal interpenetrating double-network formation, the distribution and interaction between alginate and TCNF played a crucial role. The affinity for calcium ions is higher for the guluronate unit in junction zones. Therefore, when the guluronates unit was dominant in LH alginate, the interfacial calcium ion cross-linking between TCNF and LH alginate would not be prioritized.

These composites featuring interpenetrating double networks also exhibited remarkable wet mechanical properties, as presented in Figure 6b and summarized in Table S4. The introduction of alginates resulted in a significant enhancement, elevating the wet tensile strength of the composites to over 8.6 MPa and doubling that of the neat TCNF (4.3 MPa). Similar to the observation in mechanical properties at the dry state under 50% RH, the interaction between the TCNF and AE was stronger compared to the TCNF and LH. With increasing molecular weight of the AE, the Young’s modulus of TCNF/AE composites followed an ascending trajectory from 170 MPa (145 kDa) to 275 MPa (230 kDa), reaching 430 MPa for the AE with the highest molecular weight (444 kDa). In contrast, the TCNF/LH composites exhibited only slight increases in modulus (from 135 to 183 MPa) as the molecular weight of LH increased from 107 to 267 kDa. These effects were attributed to the elongation of individual alginate chains in the composites with an increasing molecular weight. After calcium ion cross-linking, this elongation potentially contributed to the formation of a more rigid network, enhancing stiffness through interlocking between the double networks. As a result, the introduction of alginate improved the water stability of the TCNF network. The TCNF/AE1 composite with the highest molecular weight of alginate exhibited the lowest thickness swelling in water, underscoring the improved network stiffness at the wet state.

### 3.4 TCNF/AE1 Composite as a Spray Coating for Packaging Application

**Figure 7:**
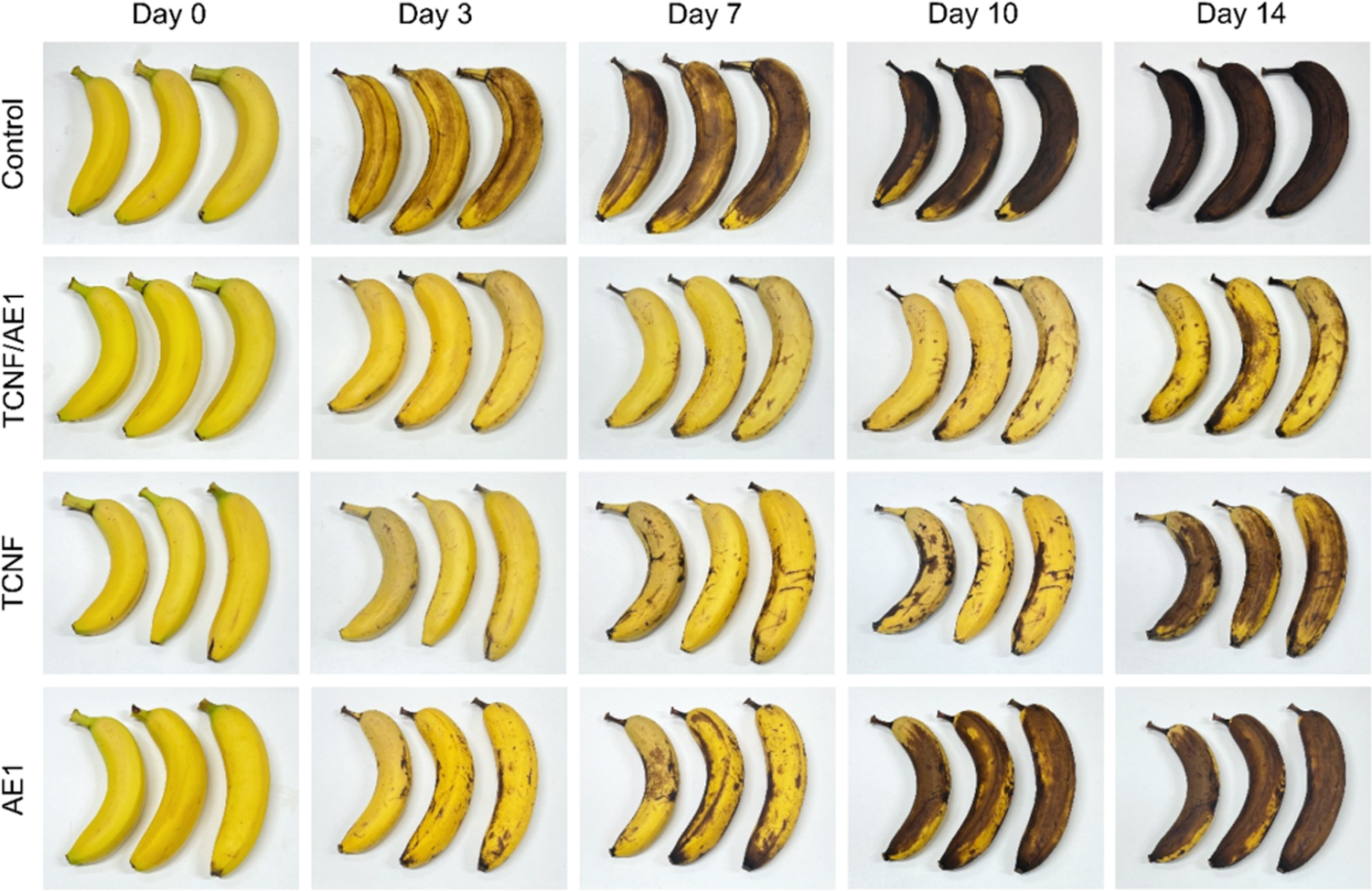
Effect of TCNF/AE1 composite spray coating on the visual appearance of bananas over time under ambient conditions (22 °C. 30% RH), in comparison with the uncoated, neat TCNF-coated, and neat AE1-coated bananas.

Due to the enhanced density and exceptional mechanical properties resulting from the superior double network of TCNF/alginates composites, we conducted a practical demonstration showcasing their potential as protective barrier films in food packaging application. Based on the mechanical performance and water stability, the TCNF/AE1 composite was used in this demonstration. The TCNF/AE1 water suspension was spray-coated onto fresh bananas, followed by Ca^2+^ cross-linking to form a barrier coating for preserving fruit freshness. The surfaces of the uncoated banana and the bananas coated with neat TCNF, neat AE1, and TCNF/AE1 were characterized by FE-SEM, as shown in Figure S5. Neat TCNF, neat AE1, and TCNF/AE1 were successfully deposited onto banana peels as barrier coatings, while the most uniform coating was achieved by TCNF/AE1. The effect of the TCNF/AE1 composite coating on the visual appearance of bananas over 2 weeks was recorded and compared to the uncoated, neat TCNF-coated, and neat AE1-coated bananas (Figure 7). The weight loss over the 2 weeks of exposure under ambient conditions is summarized in Figure S6. For uncoated bananas, obvious enzymatic browning on the surface^49^ already occurred after 3 days, while the peel turned almost completely black at day 10. The uncoated bananas eventually suffered from a weight loss of 37.4% on day 14. With the TCNF/AE1 coating, the browning was delayed considerably, while the weight loss until day 14 was remarkably lower, only 16.8%. Neat TCNF also alleviated the browning compared to the uncoated controls, similar to previous report, where bleached CNF from carrot was applied as a spray coating.^50^ However, a more severe browning was observed after day 10, and the weight loss after 14 days was 24.7%, higher than that of the TCNF/AE1-coated samples. The mechanism of delaying browning was probably mainly caused by the surface coating protection against moisture and oxygen, so that the respiration rate and the production of ethylene from the fruit were reduced.^49,50^ As a result, neat AE1 and neat TCNF coating could not provide a comparable barrier effect as the TCNF/AE1 composite coating with an interpenetrating double network, where the interfacial cross-linking enhances the network density and stiffness. Indeed, the oxygen permeability of the TCNF/AE1 composite film was 0.126 cm^3^·μm·m^–2^·kPa^–1^·day^–1^, while the neat TCNF film presented a higher value of 0.295 cm^3^·μm·m^–2^·kPa^–1^·day^–1^. In addition, the water vapor transmission rate for the TCNF/AE1 composite film was 4.94 ± 0.11 g h^–1^ m^–2^, even lower than that of neat TCNFs (6.22 ± 0.12 g h^–1^ m^–2^). The weight loss of the bananas was mainly attributed to moisture loss during storage, where a denser barrier coating was desirable, as it could provide a more effective barrier with a lower water vapor transmission rate.^51^

**Figure 8:**
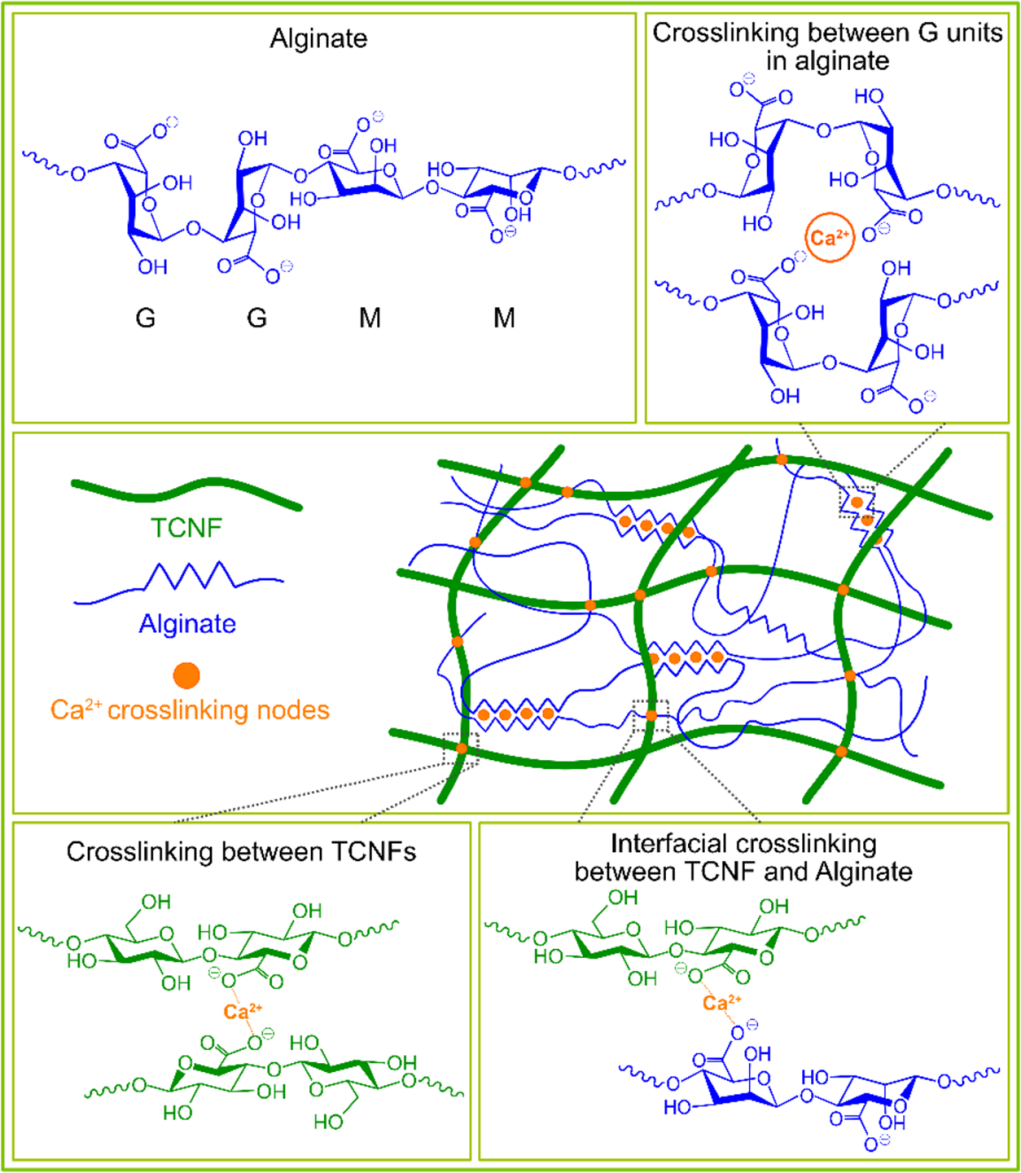
Schematic illustration of the interpenetrating double-network formation in the TCNF/alginate composite showing different calcium ion cross-linking mechanisms.

Figure 8 presents a schematic illustration for the formation of an interpenetrating double network in the TCNF/alginate composite, highlighting the synergistic effects from interfacial cross-linking, where calcium ions not only connect adjacent TCNF nanofibers and interact with guluronate units in junction zones but also impart bonding at the interfaces. This interfacial cross-linking significantly contributed to the robustness of the TCNF/alginate composite network, resulting in synergistically enhanced network density, stiffness, strength, and water stability. In the EnzyCNF/alginate and QCNF/alginate composites, an interpenetrating double network might be formed, but the absence of interfacial cross-linking between the CNFs and alginate networks compromised the performance of the composites.

## 4 Conclusions

In summary, this research underscores the importance of surface charge modification in CNFs for establishing strong interactions with alginates. The formation of an interpenetrating double network with interfacial cross-links via calcium ion cross-linking between alginate and carboxylate groups on CNF surfaces is essential for the synergistic enhancement in mechanical properties for the CNF/alginate composite. The G/M ratio and molecular weight of alginate are also important. Alginate with a higher molecular weight and a lower G/M ratio showed stronger interactions with TCNF, resulting in composites with improved mechanical performance and water stability. We demonstrated that the TCNF/AE1 composite spray coating is effective in delaying the enzymatic browning of banana peels and alleviating the weight loss of bananas, offering significant potential for the food packaging industry to maintain product freshness and quality under ambient conditions.

## Supporting Information Available

XRD patterns and monosaccharide composition of the TCNF, EnzyCNF, QCNF, and starting pulp fibers; FTIR spectra of CNFs and their respective composites with SA; FE-SEM images of film surfaces of different CNFs and their respective composites with SA; mechanical property data of the neat CNFs and CNF/SA composites at RH50%; typical stress–strain curves for the SA, AE, and LH alginate films at RH50%; mechanical property data of TCNF/AE and TCNF/LH composites at RH50% and wet state; FE-SEM images of the banana surface of uncoated control, and banana surfaces coated with TCNF/AE1, TCNF, and AE1; weight loss over time of TCNF/AE1-coated bananas compared with uncoated control, neat TCNF-coated, and neat AE1-coated bananas. (PDF)
