# Supplementary material for: Cellulose Nanofibrils/Alginates Double-Network Composites: Effects of Interfibrillar Interaction and G/M Ratio of Alginates on Mechanical Performance
Source: Biomacromolecules. 2024 Jul 8;25(8):4797–808. doi: 10.1021/acs.biomac.4c00093 (PMC11323017; doi:10.1021/acs.biomac.4c00093)
Supplement: Supplementary file 1 — bm4c00093_si_001.pdf [file bm4c00093_si_001.pdf]

Supporting Information

Cellulose nanofibrils/alginates double network composites: effects of interfibrillar interaction and G/M ratio of alginates on mechanical performance

*Li Zha [a], Finn Lillelund Aachmann [b], Håvard Sletta [c],Øystein Arlov [c], Qi Zhou [a,]**

[a] Division of Glycoscience, Department of Chemistry, School of Engineering Sciences in Chemistry, Biotechnology and Health, KTH Royal Institute of Technology, AlbaNova University Centre, SE-106 91 Stockholm, Sweden

[b] Norwegian Biopolymer Laboratory (NOBIPOL), Department of Biotechnology and Food Science, NTNU Norwegian University of Science and Technology, Sem Sælands vei 6/8, 7491 Trondheim, Norway

[c] Department of Biotechnology and Nanomedicine, SINTEF Industry, Richard Birkelands vei 3 B, 7034 Trondheim, Norway

* Corresponding author. Tel: +46 8 790 96 25, e-mail: qi@kth.se

This PDF file of Supporting Information includes:

Supplementary Figures S1 to S6

Supplementary Tables S1 to S4

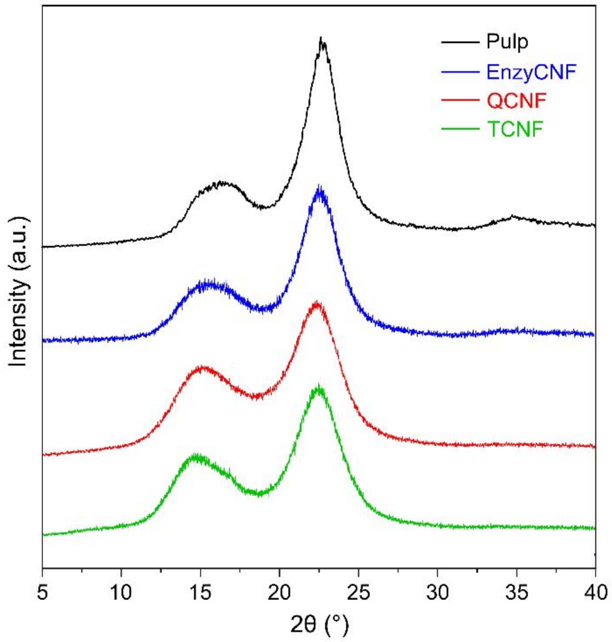

Figure S1. X-ray diffraction (XRD) patterns of the TCNF, EnzyCNF, and QCNF samples as compared to the starting pulp fibers.

Table S1. Monosaccharide composition of pulp fibers, TCNF, EnzyCNF and QCNF

Samples	Arabinose (%)	Galactose (%)	Glucose (%)	Xylose (%)	Mannose (%)	Cellulose (%)	Hemicellulose (%)
Pulp	0.2	0.2	84.7	6.4	8.5	81.9	18.1
TCNF	0.1	0.1	91.9	3.9	4.0	90.6	9.4
EnzyCNF	0.1	0.2	88.2	4.9	6.6	86.0	14.0
QCNF	0.1	0.1	97.2	1.3	1.3	96.8	3.2

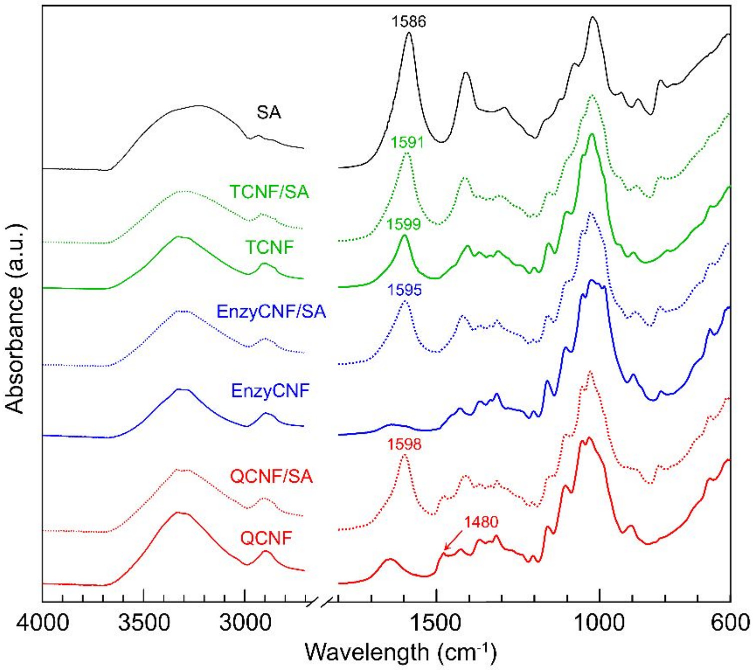

Figure S2. FTIR spectra of different CNFs and their respective composites with SA

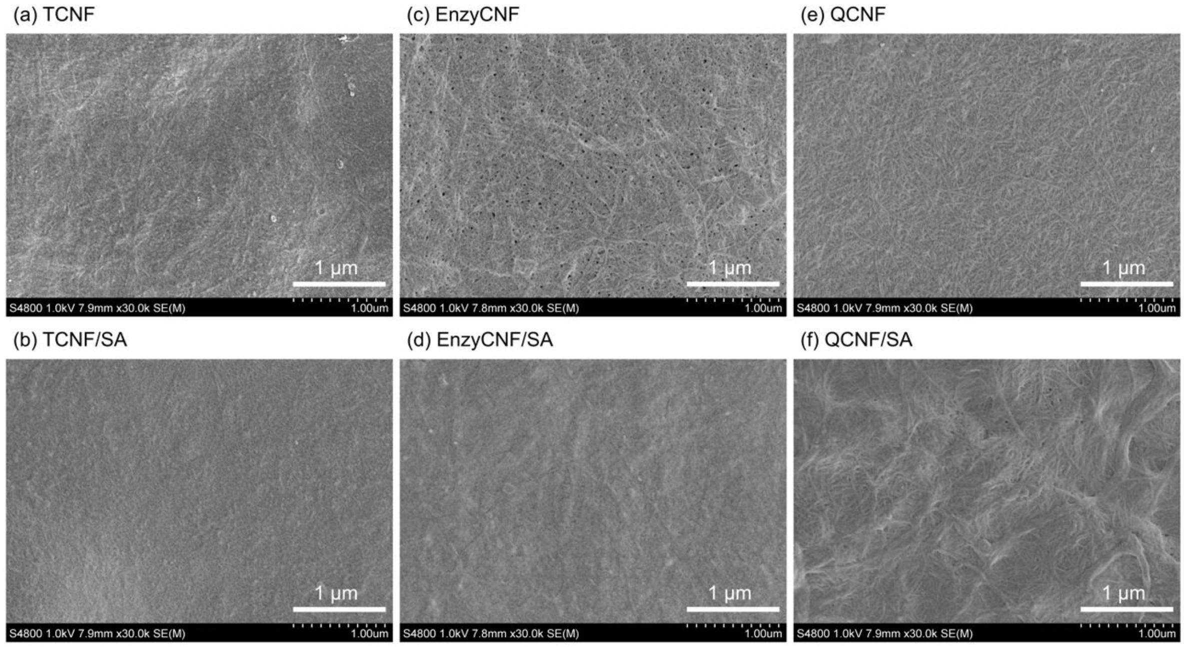

Figure S3. SEM images of film surfaces of different CNFs and their respective composite with SA.

Table S2. Physical and mechanical properties of the neat CNFs and the CNF/SA composites at RH50% including tensile strength (σ), strain to failure (ε), Young's modulus (*E*), yield strength (σ_y), work to fracture, density (ρ), moisture content, specific strength and modulus.[a]

Samples	σ (MPa)	ε (%)	*E* (GPa)	σ_y (MPa)	Work to fracture (MJ/m³)	ρ (g/cm³)	Moisture content (wt.%)	Specific strength (kNm/kg)	Specific modulus (MNm/kg)
SA	115 (5)	1.8 (0.1)	8.5 (0.5)	98 (3)	1.3 (0.1)	1.47	16.8	78.2	5.8
QCNF	158 (9)	9.6 (0.6)	7.3 (0.3)	51 (3)	10.2 (0.4)	1.32	13.3	119.7	5.5
QCNF/SA	152 (9)	5.3 (0.4)	8.3 (0.4)	90 (4)	5.7 (0.2)	1.34	10.9	113.4	6.2
EnzyCNF	295 (9)	7.7 (0.5)	10.4 (0.3)	120 (4)	14.9 (0.4)	1.37	7.6	215.3	7.6
EnzyCNF/SA	250 (10)	8.1 (0.4)	9.3 (0.4)	105 (3)	13.2 (0.3)	1.38	7.7	181.2	6.7
TCNF	300 (11)	6.2 (0.3)	14.3 (0.7)	118 (3)	11.6 (0.5)	1.41	9.3	212.8	10.1
TCNF/SA	327 (12)	3.4 (0.2)	20.0 (1.0)	166 (4)	7.2 (0.5)	1.48	7.3	220.9	13.5

[a] The values in parentheses are the sample standard deviations.

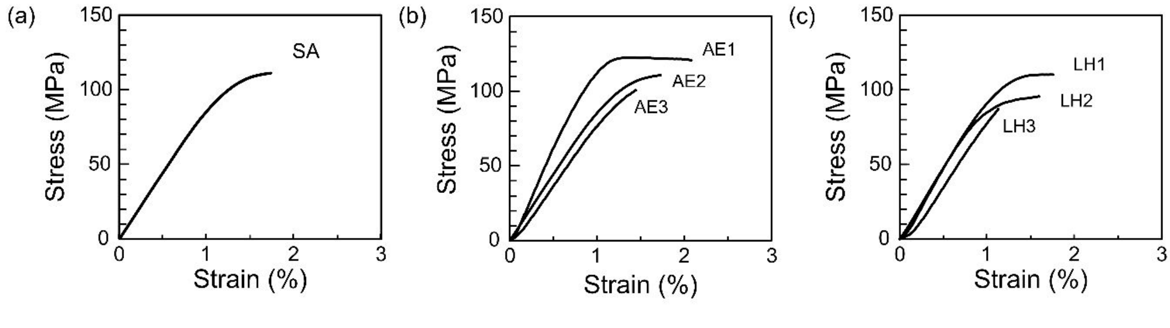

Figure S4. Typical tensile stress-strain curves for the films of the SA, AE, and LH alginates at RH 50%.

Table S3. Physical and mechanical properties of TCNF/AE and TCNF/LH composites at RH50% including tensile strength (σ), strain to failure (ε), Young's modulus (*E*), yield strength (σ_y), work to fracture, density (ρ), moisture content, specific strength and modulus.[a]

Samples	σ (MPa)	ε (%)	*E* (GPa)	σ_y (MPa)	Work to fracture (MJ/m^3)	ρ (g/cm^3)	Moisture content (wt.%)	Specific strength (kNm/kg)	Specific modulus (MNm/kg)
AE1	122 (5)	2.1 (0.2)	12.4 (0.4)	110 (5)	1.9 (0.3)	1.48	16.3	82.4	8.4
AE2	109 (4)	1.7 (0.1)	8.7 (0.3)	96 (4)	1.2 (0.1)	1.46	17.8	74.7	6.0
AE3	100 (4)	1.4 (0.1)	7.8 (0.3)	--	0.7 (0.1)	1.45	18.4	69.0	5.4
LH1	108 (5)	1.7 (0.2)	9.4 (0.3)	99 (3)	1.2 (0.2)	1.46	18.3	74.0	6.4
LH2	95 (3)	1.5 (0.2)	9.3 (0.2)	81 (3)	1.0 (0.1)	1.46	18.6	65.1	6.4
LH3	87 (4)	1.1 (0.1)	7.7 (0.2)	--	0.5 (0.1)	1.44	19.2	60.4	5.3
TCNF/ AE1	331 (9)	2.8 (0.3)	20.3 (0.7)	208 (7)	5.9 (0.3)	1.49	7.0	222.1	13.6
TCNF/ AE2	312 (8)	3.3 (0.2)	19.5 (0.7)	156 (6)	6.7 (0.4)	1.48	7.4	210.8	13.2
TCNF/ AE3	316 (10)	4.1 (0.3)	17.7 (0.5)	147 (5)	8.5 (0.4)	1.45	7.8	217.9	12.2
TCNF/ LH1	293 (11)	3.9 (0.4)	17.3 (0.6)	142 (6)	7.5 (0.5)	1.45	7.9	202.1	11.9
TCNF/ LH2	259 (8)	3.4 (0.2)	14.7 (0.4)	137 (4)	5.8 (0.3)	1.44	8.2	179.9	10.2
TCNF/ LH3	220 (7)	2.5 (0.2)	13.9 (0.3)	136 (6)	3.5 (0.2)	1.44	8.2	152.8	9.7

[a] The values in parentheses are the sample standard deviations.

Table S4. Physical and mechanical properties of TCNF/AE and TCNF/LH composites at wet state including tensile strength (σ), strain to failure (ε), Young's modulus (*E*), work to fracture, water content and thickness swelling.[a]

Samples	σ (MPa)	ε (%)	*E* (MPa)	Work to fracture (MJ/m^3)	Water content (wt.%)	Thickness swelling (%)
TCNF	4.3 (0.3)	22.4 (2.1)	89 (4)	0.65 (0.06)	72	339
TCNF/SA	9.5 (0.3)	15.5 (1.2)	295 (25)	0.93 (0.05)	71	212
TCNF/AE1	9.3 (0.4)	9.3 (0.7)	430 (32)	0.63 (0.04)	69	145
TCNF/AE2	9.7 (0.5)	19.5 (1.6)	275 (23)	1.17 (0.11)	71	233
TCNF/AE3	8.7 (0.3)	26.5 (2.4)	170 (13)	1.53 (0.20)	72	239
TCNF/LH1	8.8 (0.4)	23.2 (2.1)	183 (10)	1.25 (0.12)	72	240
TCNF/LH2	8.8 (0.5)	24.8 (2.0)	155 (12)	1.27 (0.15)	72	245
TCNF/LH3	8.6 (0.4)	29.0 (2.2)	135 (11)	1.40 (0.11)	73	258

[a] The values in parentheses are the sample standard deviations.

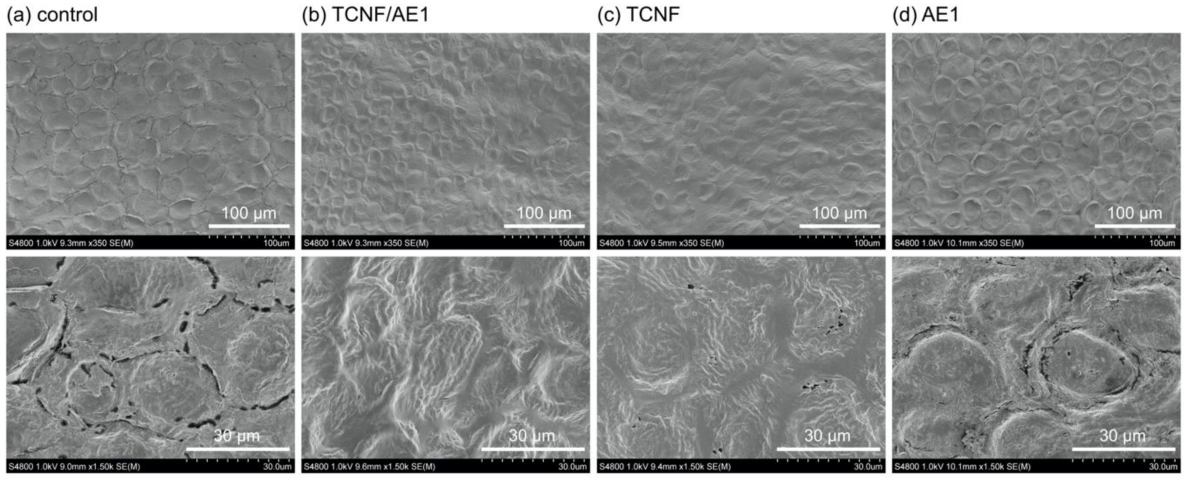

Figure S5. SEM images of banana surface of (a) uncoated control, compared with banana surfaces coated with (b) TCNF/AE1, (c) TCNF, and (d) AE1.

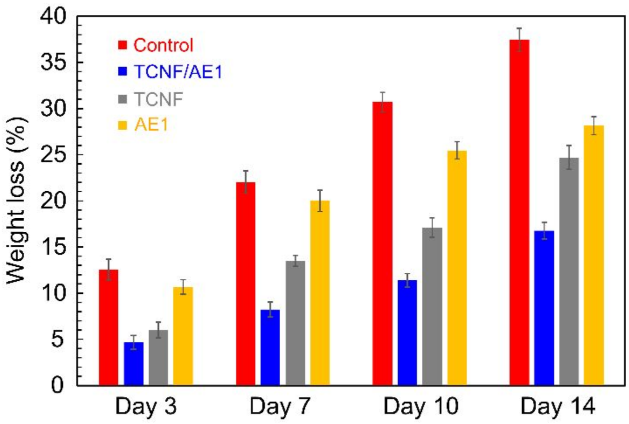

Figure S6. Effect of the TCNF/AE1 composite spray coating on the weight loss of bananas over time under ambient condition (22˚C, RH30%), in comparison with uncoated control, neat TCNF coated, and neat AE1 coated bananas.
